# Overexpression of METTL3 attenuates high-glucose induced RPE cell pyroptosis by regulating miR-25-3p/PTEN/Akt signaling cascade through DGCR8

**DOI:** 10.18632/aging.103130

**Published:** 2020-05-04

**Authors:** Xu Zha, Xiaoting Xi, Xinyu Fan, Minjun Ma, Yuanping Zhang, Yanni Yang

**Affiliations:** 1 Department of Ophthalmology, The 2nd Affiliated Hospital of Kunming Medical University, Kunming Yunnan, China; 2 Department of Ophthalmology, The First Affiliated Hospital of Kunming Medical University, Kunming Yunnan, China

**Keywords:** METTL3, miR-25-3p, m6A, pyroptosis, PTEN

## Abstract

Methyltransferase-like protein 3 (METTL3) regulates multiple cell functions and diseases by modulating N^6^-methyladenosine (m^6^A) modifications. However, it is still unclear whether METTL3 involves in the pathogenesis of diabetic retinopathy (DR). In the present study, we found that high-glucose inhibited RPE cell proliferation, promoted cell apoptosis and pyroptosis in a time-dependent manner. In addition, both METTL3 mRNA and miR-25-3p were low-expressed in the peripheral venous blood samples of diabetes mellitus (DM) patients compared to normal volunteers, and high-glucose inhibited METTL3 and miR-25-3p expressions in RPE cells. As expected, upregulation of METTL3 and miR-25-3p alleviated the cytotoxic effects of high-glucose on RPE cells, and knock-down of METTL3 and miR-25-3p had opposite effects. Additionally, METTL3 overexpression increased miR-25-3p levels in RPE cells in a microprocessor protein DGCR8-dependent manner, and miR-25-3p ablation abrogated the effects of overexpressed METTL3 on cell functions in high-glucose treated RPE cells. Furthermore, PTEN could be negatively regulated by miR-25-3p, and overexpression of METTL3 increased phosphorylated Akt (p-Akt) levels by targeting miR-25-3p/PTEN axis. Consistently, upregulation of PTEN abrogated the protective effects of METTL3 overexpression on RPE cells treated with high-glucose. Collectively, METTL3 rescued cell viability in high-glucose treated RPE cells by targeting miR-25-3p/PTEN/Akt signaling cascade.

## INTRODUCTION

Post-transcriptional modifications are closely associated with the development of multiple diseases and cell functions [1–3]. Currently, more than 100 types of RNA modifications have been identified [4]. Among them, N^6^-methyladenosine (m^6^A) was the most common RNA modifications [5], which participated in the pathogenesis of type 2 diabetes mellitus (T2DM) [6] and its complications [7], such as diabetic retinopathy (DR) [7]. Methyltransferase-like 3 (METTL3) served as a m^6^A modification “writer”, which was critical for regulating m^6^A modifications [8]. Recent study validated that glucose regulated METTL3 mediated m^6^A modifications in T2DM [6] and diabetic cataract [9]. However, the role of METTL3 in the regulation of diabetes associated diseases is controversial [6, 9], and the association between METTL3 and DR pathogenesis is still largely unknown. In addition, retinal pigment epithelium (RPE) cells were the main cells of the retina and widely used as an *in vitro* cellular model for DR research [10], hence, the RPE cell line ARPE-19 was selected in this study according to the previous publication [11].

Aside from messenger RNA (mRNA) [12], ribosomal RNA (rRNA) [13] and transfer RNA (tRNA) [14], METTL3 mediated m^6^A modifications regulated the expression levels of non-coding RNA, such as Long non-coding RNAs (LncRNAs) [15], circular RNAs (CircRNAs) [16] and microRNAs (miRNAs) [17]. Specifically, recent data indicated that METTL3 promoted the maturation of multiple miRNAs, including let-7e, miR-221/222, miR-4485, miR-25-3p, miR-93, miR-126 and miR-335, in a m^6^A dependent manner [4, 18]. Interestingly, our preliminary experiments screened out that miR-25-3p, instead of other miRNAs, was significantly downregulated in high-glucose treated RPE cells compared to the control group. MiR-25-3p was reported to regulate cell proliferation [19, 20] and death [20]. Mechanistically, miR-25-3p promoted glioma cell proliferation by targeting FBXW7 as well as DKK3 [19], and inhibited breast cancer cell apoptosis by targeting BTG2 [20]. Notably, miR-25-3p modulated retinal degeneration [21] and attenuated high-glucose induced cell apoptosis [21].

Phosphatase and tensin homolog (PTEN) was identified as a tumor suppressor and inhibited the development of multiple cancers [22–24]. Aside from cancers, recent studies also validated that PTEN was closely related with diabetes mellitus [25, 26] and DR progression [27]. For example, high-glucose induced human umbilical vein endothelial cells (HUVECs) death by upregulating PTEN [28]. In addition, high-glucose promoted epithelial-mesenchymal transition (EMT) in human mesothelial peritoneal cells by modulating PTEN [29], and upregulation of PTEN inhibited retinal vascular endothelial cell growth by inactivating PI3K/Akt signal pathway [27]. Notably, PTEN/Akt axis was the downstream target of miR-25-3p [30] and overexpressed miR-25-3p alleviated high-glucose induced renal tubular epithelial cell death by inactivating PTEN/Akt signal pathway [31].

Collectively, this study aimed to investigate the involvement of METTL3 mediated m^6^A modifications in the regulation of DR pathogenesis, and uncover the underlying mechanisms. This study will shed light on the discovery of potential therapeutic agents for DR treatment in clinic.

## RESULTS

### The expression levels of METTL3 and miR-25-3p in clinical samples and RPE cells

**Figure 1:**
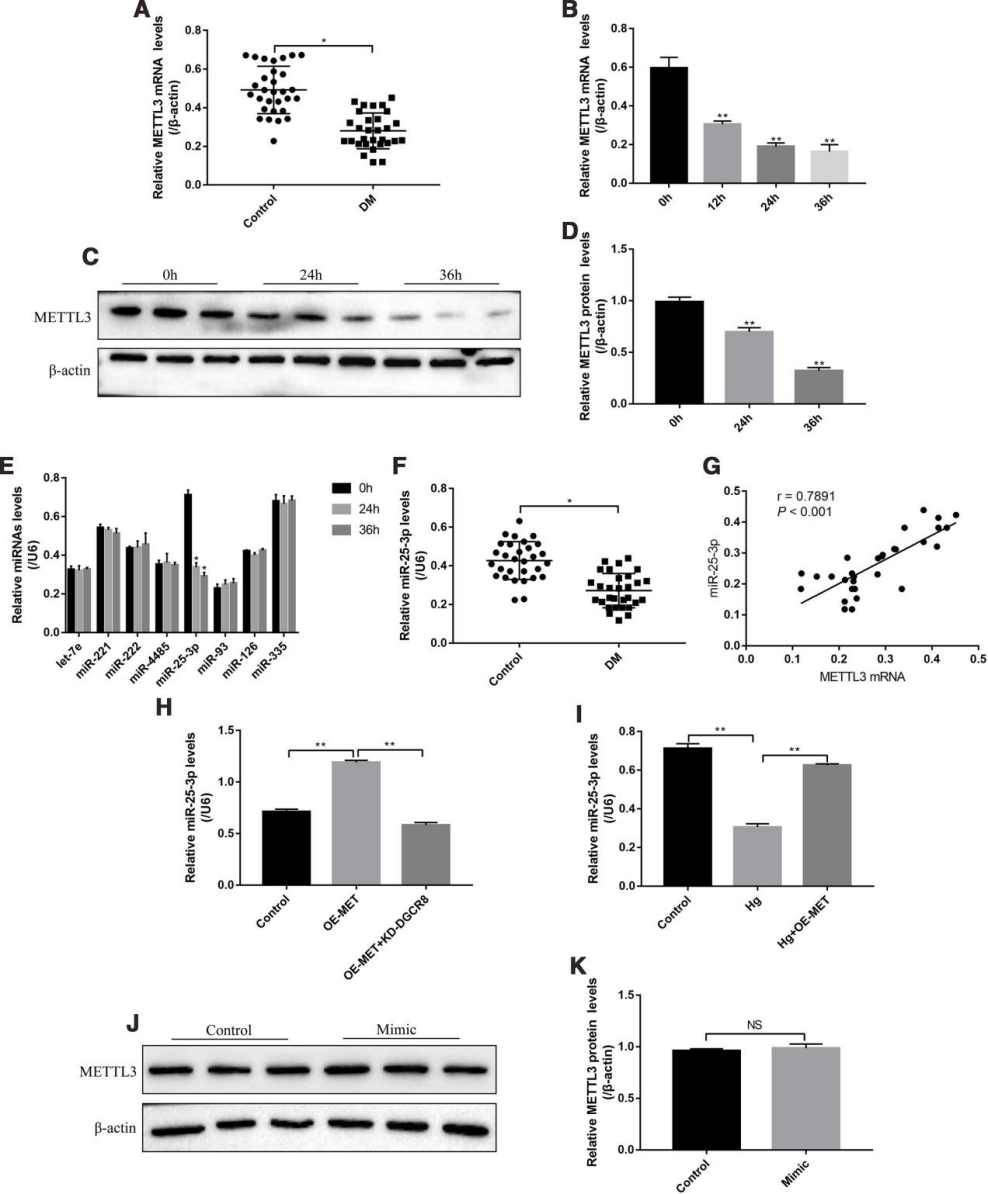
**The expression status of METTL3 and miR-25-3p in T2DM clinical samples and RPE cells.** Real-Time qPCR was employed to determine the levels of METTL3 mRNA in (**A**) clinical serum samples and (**B**) RPE cells treated with high-glucose for 0 h, 12 h, 24 h and 36 h, respectively. (**C**, **D**) Western Blot was conducted to determine the expression levels of METTL3 in RPE cells treated with high-glucose for 0 h, 24 h and 36 h respectively. (**E**) RPE cells were treated with high-glucose for 0 h, 24 h and 36 h, respectively, the levels of let-7e, miR-221, miR-222, miR-4485, miR-25-3p, miR-93, miR-126 and miR-335 were screened by Real-Time qPCR. (**F**) The levels of miR-25-3p were measured by Real-Time qPCR in clinical samples. (**G**) The correlations of miR-25-3p and METTL3 mRNA in the clinical specimens collected from T2DM patients were determined by using the Pearson Correlation Analysis. (**H**, **I**) The levels of miR-25-3p were determined by Real-Time qPCR. (**J**, **K**) Western Blot was performed to detect the expression status of METTL3 in RPE cells. Each experiment had at least 3 repetitions, the data were collected and represented as Mean ± SD. “*” means *p* < 0.05 and “**” means *p* < 0.01.

The patients (N=30) diagnosed with type II diabetes mellitus (T2DM) and healthy volunteers (N=30) were recruited, and their peripheral venous blood samples were collected as the experimental group (DM groups) and control group, respectively. The results showed that METTL3 mRNA was low-expressed in T2DM groups comparing to the control group (Figure 1A). In addition, the RPE cells were treated with high-glucose (50 mM) for 0h, 12h, 24h and 36h according to our previous study [32]. The results showed that high-glucose decreased the expression levels of METTL3 in a time-dependent manner (Figure 1B–1D). METTL3 potentially regulated multiple miRNAs (let-7e, miR-221, miR-222, miR-4485, miR-25-3p, miR-93, miR-126 and miR-335) [4, 18], and we identified that high-glucose specifically inhibited the levels of miR-25-3p, instead of other miRNAs, in RPE cells (Figure 1E). Similarly, the levels of miR-25-3p were lower the peripheral venous blood samples collected from T2DM patients compared to the normal volunteers (Figure 1F). In parallel, the levels of METTL3 mRNA and miR-25-3p positively correlated in T2DM patients clinical samples (Figure 1G). Further results showed that overexpressed METTL3 increased miR-25-3p levels in RPE cells, which were abrogated by knocking down DGCR8 (Figure 1H), indicating that METTL3 promoted miR-25-3p expressions in a DGCR8-dependent manner [18]. Furthermore, the inhibiting effects of high-glucose on miR-25-3p levels were abrogated by overexpressing METTL3 (Figure 1I), but miR-25-3p overexpression had little effects on METTL3 in RPE cells (Figure 1J, 1K).

### The effects of METTL3 on cell proliferation, apoptosis and pyroptosis in high-glucose treated RPE cells

**Figure 2:**
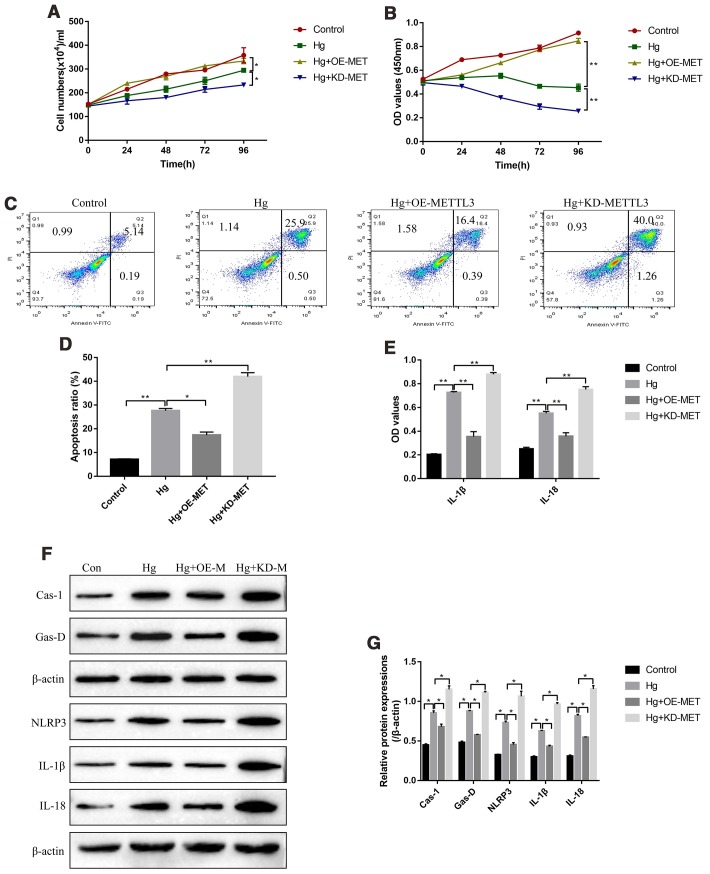
**METTL3 affected high-glucose regulated RPE cell proliferation, apoptosis and pyroptosis.** (**A**) Cell counting assay was employed to measure RPE cell division abilities. (**B**) CCK-8 assay was conducted to determine RPE cell proliferation abilities. (**C**, **D**) FCM was performed to detect RPE cell apoptosis ratio. (**E**) ELISA was performed to measure the expression levels of IL-1β and IL-18 in the supernatants of RPE cells. (**F**, **G**) Western Blot was used to determine the expression status of pyroptosis associated proteins (Caspase-1, Gasdermin D, NLRP3, IL-1β and IL-18) in RPE cells. (“Hg” means “High-glucose”, “OE-M” means “Overexpressed METTL3” and “KD-M” means “Knock-down of METTL3”). Each experiment had at least 3 repetitions, the data were collected and represented as Mean ± SD. “*” means *p* < 0.05 and “**” means *p* < 0.01.

Further experiments were conducted to explore the effects of METTL3 on RPE cell functions, such as cell proliferation, apoptosis and pyroptosis. The cell counting assay results showed that high-glucose inhibited RPE cell division, which were aggravated by knocking down METTL3 and reversed by overexpressing METTL3 (Figure 2A). Similarly, the CCK-8 assay results evidenced that the inhibiting effects of high-glucose on RPE cell proliferation were enhanced by downregulating METTL3 and restored by upregulating METTL3 (Figure 2B). In parallel, the FCM results showed that overexpression of METTL3 alleviated, while knock-down of METTL3 enhanced high-glucose induced RPE cell apoptosis (Figure 2C, 2D). Of note, METTL3 involved in the regulation of high-glucose induced RPE cell pyroptosis. Mechanistically, the ELISA results showed that high-glucose increased the expression levels of IL-1β and IL-18 in the supernatants of RPE cells, which were decreased by overexpressing METTL3 and increased by knocking down METTL3 (Figure 2E). Consistently, the Western Blot results showed that high-glucose induced upregulation of pyroptosis associated proteins (Caspase-1, Gasdermin D, NLRP3, IL-1β and IL-18) were alleviated by overexpressing METTL3 and aggravated by knocking down METTL3 in RPE cells (Figure 2F, 2G).

### The effects of miR-25-3p on the cell functions in RPE cells treated with high-glucose

**Figure 3:**
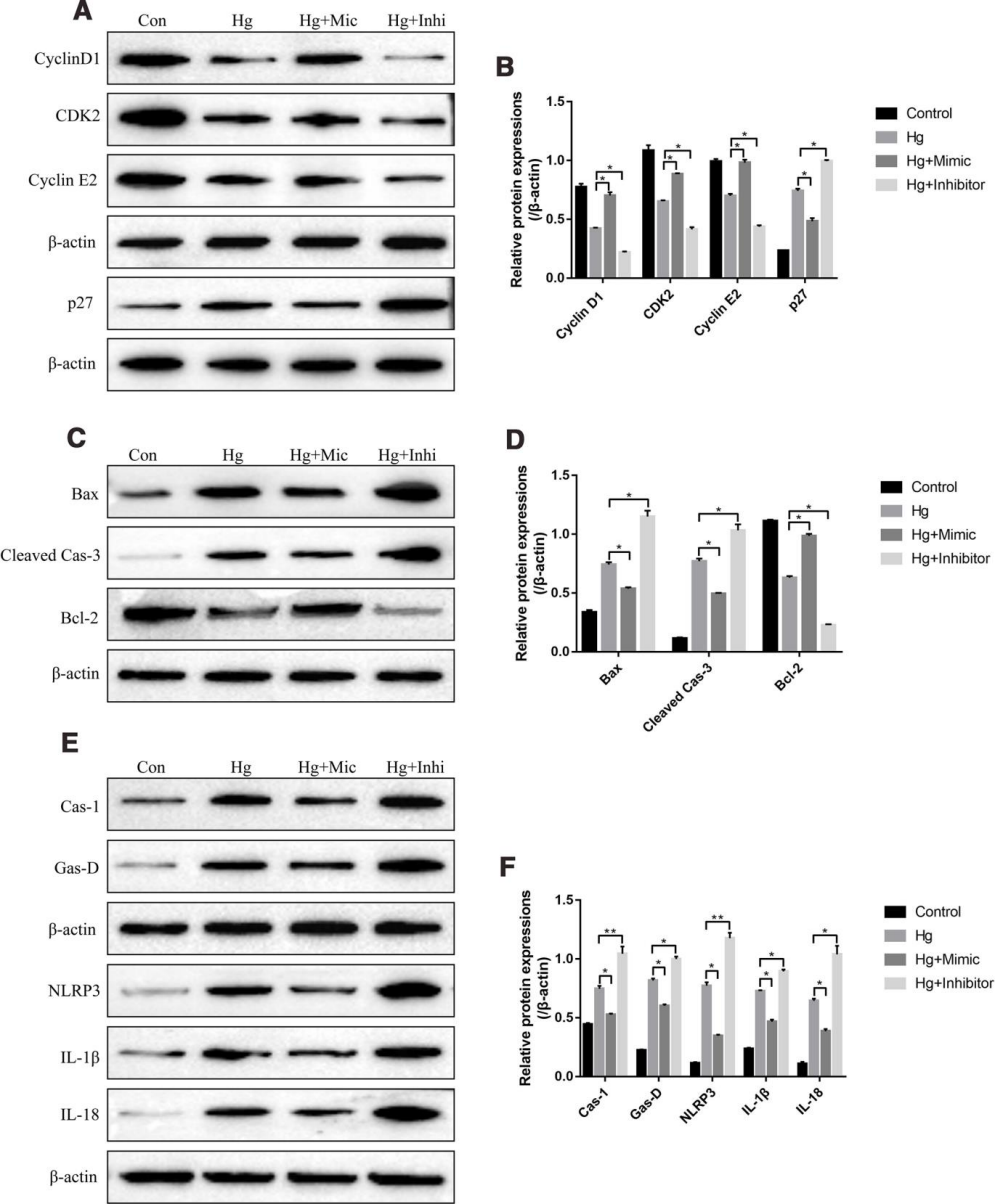
**High-glucose regulated RPE cell functions by downregulating miR-25-3p.** Western Blot was conducted to determine the expressions of (**A**, **B**) proliferation associated proteins (Cyclin D1, CDK2 and Cyclin E2), (**C**, **D**) apoptosis associated proteins (Bax, cleaved caspase-3 and Bcl-2) and (**E**, **F**) pyroptosis associated proteins (Caspase-1, Gasdermin D, NLRP3, IL-1β and IL-18) in RPE cells. (“Con” means “Control”, “Hg” means “High-glucose”, “Mic” means “miR-25-3p mimic” and “Inhi” means “miR-25-3p inhibitor”). Each experiment had at least 3 repetitions, the data were collected and represented as Mean ± SD. “*” means *p* < 0.05 and “**” means *p* < 0.01.

We next investigated the role of miR-25-3p in the regulation of high-glucose induced RPE cell proliferation, apoptosis and pyroptosis. To achieve this, the proliferation associated proteins (Cyclin D1, CDK2 and Cyclin E2), apoptosis associated proteins (Bax, cleaved Caspase-3 and Bcl-2) and pyroptosis associated proteins (Caspase-1, Gasdermin D, NLRP3, IL-1β and IL-18) were determined by Western Blot. The results showed that high-glucose significantly decreased the expression levels of Cyclin D1, CDK2 and Cyclin E2, promoted p27 expressions in RPE cells (Figure 3A, 3B). The effects of high-glucose on the above proliferation associated proteins were reversed by overexpressing miR-25-3p and aggravated by knocking down miR-25-3p (Figure 3A, 3B). Besides, high-glucose induced upregulation of Bax as well as cleaved Caspase-3, and downregulation of Bcl-2, which were also abrogated by transfecting cells with miR-25-3p mimic and aggravated by miR-25-3p inhibitor (Figure 3C, 3D). Furthermore, high-glucose induced upregulation of caspase-1, Gasdermin D, NLRP3, IL-1β and IL-18 in RPE cells, which were decreased by overexpressing miR-25-3p and increased by downregulating miR-25-3p (Figure 3E, 3F).

### Overexpressed METTL3 alleviated the cytotoxic effects of high-glucose on RPE cells by targeting miR-25-3p

**Figure 4:**
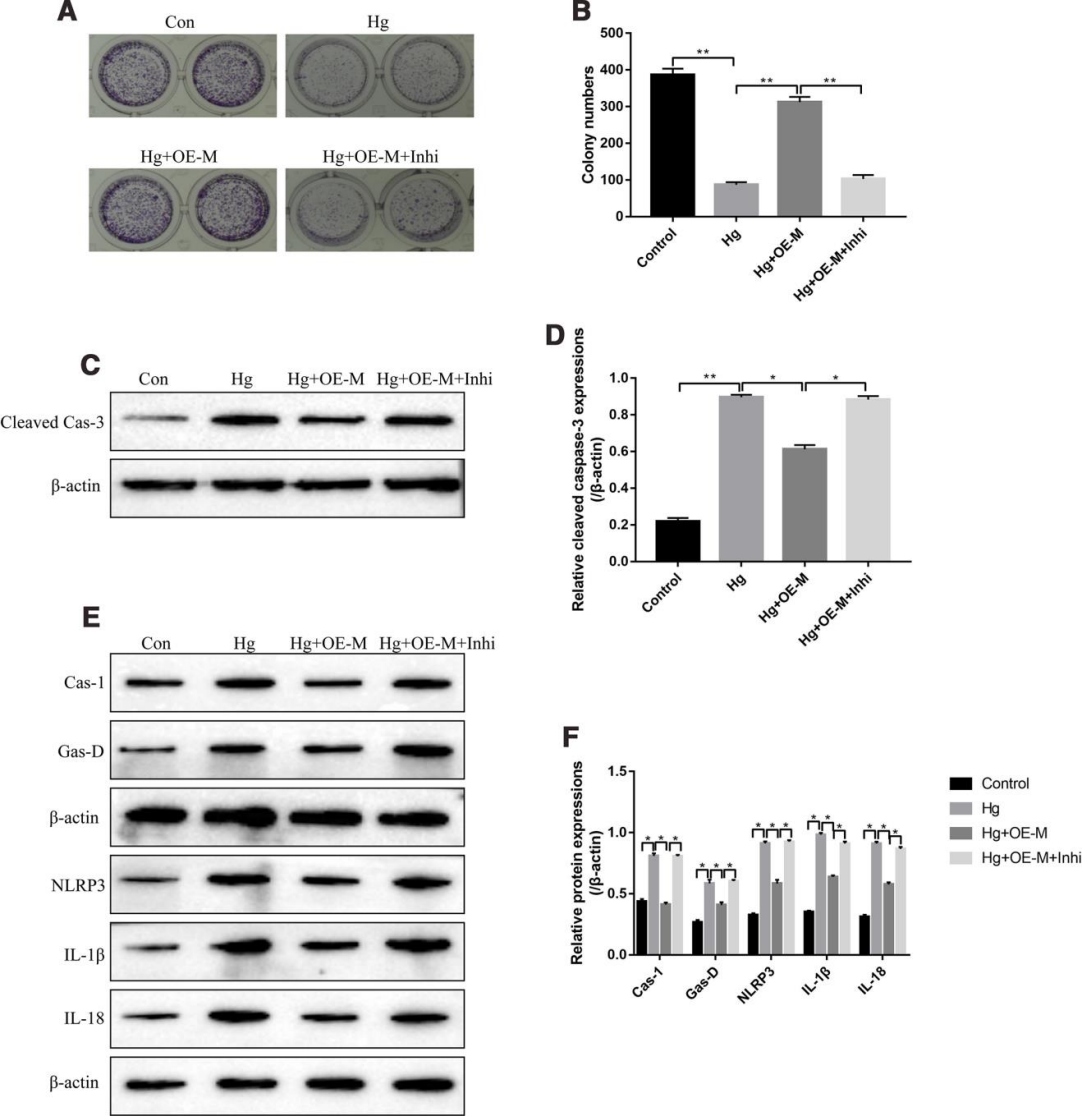
**High-glucose inhibited RPE cell viability by regulating METTL3/miR-25-3p signaling cascade.** (**A**, **B**) The colony formation assay was performed to measure RPE cell proliferation. Western Blot was used to determine the expression levels of (**C**, **D**) cleaved Caspase-3 and (**E**, **F**) pyroptosis associated proteins (Caspase-1, Gasdermin D, NLRP3, IL-1β and IL-18) in RPE cells. (“Con” means “Control”, “Hg” means “High-glucose”, “OE-M” means “Overexpressed METTL3” and “Inhi” means “miR-25-3p inhibitor”). Each experiment had at least 3 repetitions, the data were collected and represented as Mean ± SD. “*” means *p* < 0.05 and “**” means *p* < 0.01.

Based on the above findings, it was reasonable to speculate that METTL3 might participate in the regulation of high-glucose modulated cell proliferation, apoptosis and pyroptosis by targeting miR-25-3p. The colony formation assay results showed that overexpressed METTL3 alleviated the inhibiting effects of high-glucose treatment on RPE cell proliferation, which were abrogated by knocking down miR-25-3p (Figure 4A, 4B). Besides, we determined the expression status of cleaved Caspase-3 in RPE cells to evaluate cell apoptosis. The results showed that overexpression of METTL3 decreased the expression levels of cleaved Caspase-3 in high-glucose treated RPE cells, which were also reversed by transfecting cells with miR-25-3p inhibitor (Figure 4C, 4D). Furthermore, upregulation of METTL3 decreased the expression levels of pyroptosis associated proteins (Caspase-1, Gasdermin D, NLRP3, IL-1β and IL-18) in high-glucose treated RPE cells, which were also reversed by downregulating miR-25-3p (Figure 4E, 4F). The above results suggested that high-glucose inhibited RPE cell proliferation, promoted cell apoptosis and pyroptosis by regulating METTL3/miR-25-3p axis.

### METTL3 regulated PTEN/Akt signal pathway by upregulating miR-25-3p

**Figure 5:**
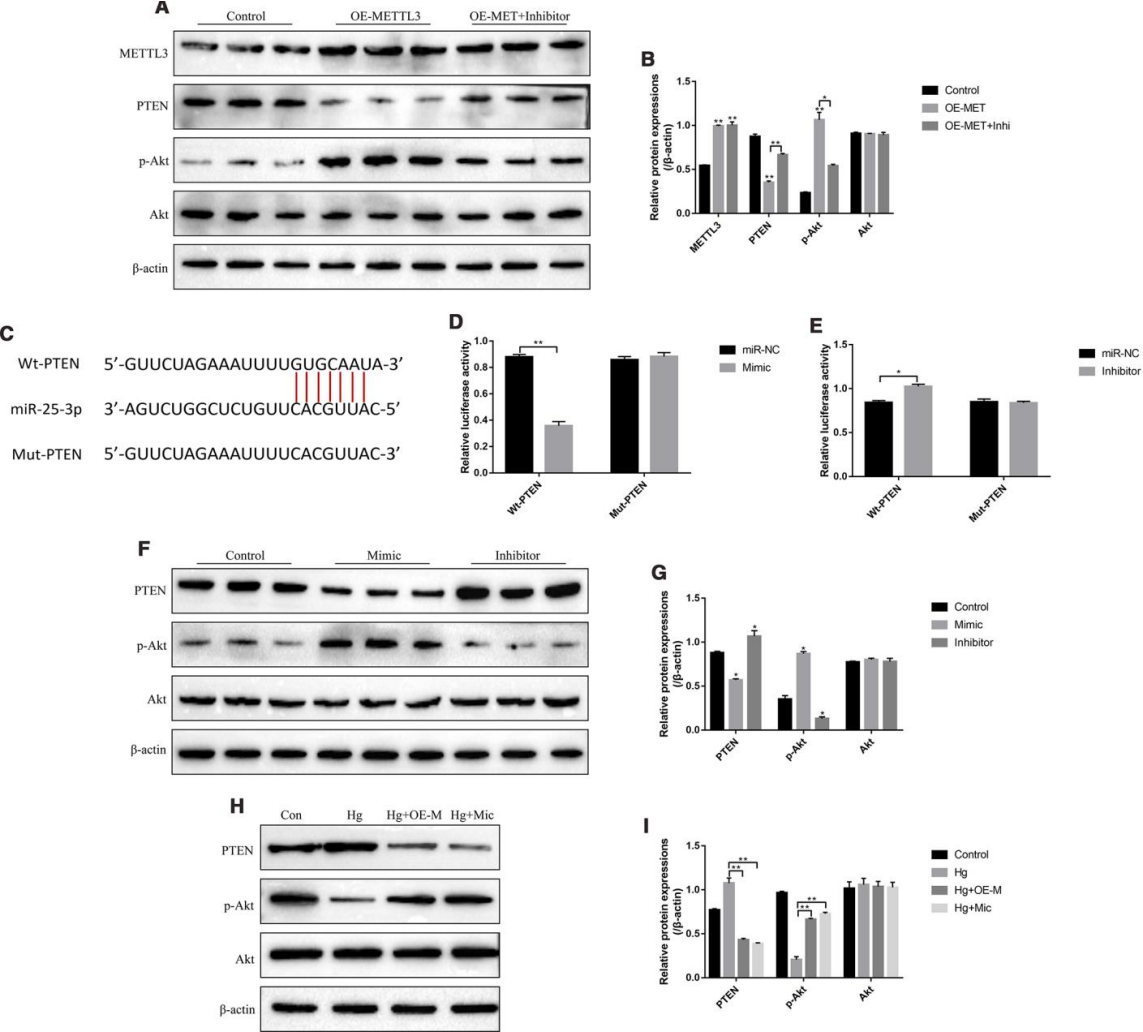
**High-glucose regulated PTEN/Akt signal pathway in RPE cells by downregulating METTL3 and miR-25-3p.** (**A**, **B**) Western Blot was used to determine the expressions of METTL3, PTEN, p-Akt and Akt in RPE cells. (**C**) The targeting sites of miR-25-3p and 3’UTR regions of PTEN mRNA were predicted by using the online starBase software. (**D**, **E**) Dual-luciferase reporter gene system was employed to validate the binding sites of miR-25-3p and 3’UTR regions of PTEN mRNA. (**F**–**I**) Western Blot was conducted to determine the expression status of PTEN, p-Akt and Akt in RPE cells. (“Con” means “Control”, “Hg” means “High-glucose”, “OE-M” means “Overexpressed METTL3” and “Mic” means “miR-25-3p mimic”). Each experiment had at least 3 repetitions, the data were collected and represented as Mean ± SD. “*” means *p* < 0.05 and “**” means *p* < 0.01.

The PTEN/Akt axis has been reported to be closely related with the development of diabetes mellitus [25, 26] and was the downstream target of miR-25-3p in high-glucose treated renal tubular epithelial cells [31], which enlightened us that high-glucose might regulate PTEN/Akt axis through METTL3 and miR-25-3p in RPE cells. As expected, the results showed that overexpressed METTL3 significantly decreased the expression levels of PTEN and increased phosphorylated Akt levels in RPE cells, which were abrogated by knocking down miR-25-3p (Figure 5A, 5B). Besides, the online starBase software predicted the targeting sites of miR-25-3p and 3’ UTR regions of PTEN mRNA (Figure 5C). The dual-luciferase reporter gene system results validated that miR-25-3p mimic decreased the luciferase activity in 293T cells (Figure 5D). Consistently, the luciferase activity was increased by transfecting cells with miR-25-3p inhibitor (Figure 5E), indicating that miR-25-3p inhibited the expression levels of PTEN by binding to its 3’ UTR regions and in accordance with the previous study [31]. Further results also validated that overexpression of miR-25-3p decreased the expression levels of PTEN and increased phosphorylated Akt in RPE cells, and downregulation of miR-25-3p had opposite effects on the above proteins (Figure 5F, 5G). Notably, we found that high-glucose increased the expression levels of PTEN and decreased phosphorylated Akt levels in RPE cells, which were all reversed by both upregulating METTL3 and miR-25-3p (Figure 5H, 5I). The above results indicated that high-glucose regulated PTEN/Akt axis by downregulating METTL3 and miR-25-3p in RPE cells.

### Upregulation of PTEN abrogated the protective effects of overexpressed METTL3 on high-glucose treated RPE cells

**Figure 6:**
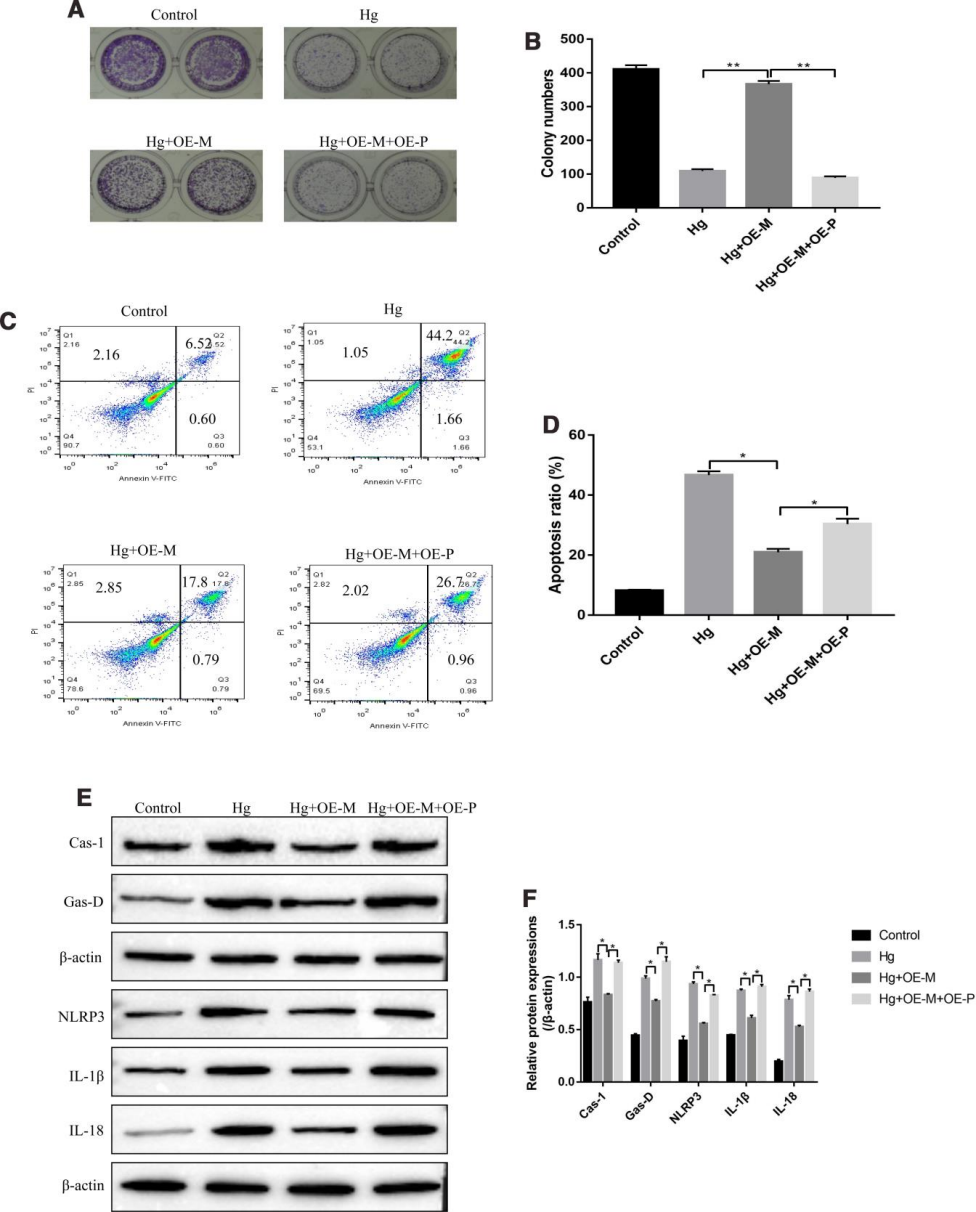
**High-glucose inhibited RPE cell viability by regulating PTEN/Akt signal pathway.** (**A**, **B**) Colony formation assay was performed to detect RPE cell proliferation. (**C**, **D**) FCM was used to determine RPE cell apoptosis ratio. (**E**, **F**) Western Blot was performed to determine the expression levels of pyroptosis associated proteins (Caspase-1, Gasdermin D, NLRP3, IL-1β and IL-18) in RPE cells. (“Hg” means “High-glucose”, “OE-M” means “overexpressed METTL3” and “OE-P” means “Overexpressed PTEN”). Each experiment had at least 3 repetitions, the data were collected and represented as Mean ± SD. “*” means *p* < 0.05 and “**” means *p* < 0.01.

The experiments were next conducted to investigate whether high-glucose regulated cell proliferation, apoptosis and pyroptosis in RPE cells by regulating METTL3/miR-25-3p/PTEN/Akt signal pathway. To achieve this, the overexpressed vectors for METTL3 and PTEN were transfected into RPE cells, respectively. The colony formation assay results showed that upregulation of METTL3 alleviated the inhibiting effects of high-glucose on RPE cell proliferation, which were abrogated by overexpressing PTEN (Figure 6A, 6B). Similarly, the FCM results showed that high-glucose significantly increased the apoptosis ratio of RPE cells, which were reversed by overexpressing METTL3. The effects of overexpressed METTL3 on high-glucose induced RPE cell apoptosis were abrogated by upregulating PTEN (Figure 6C, 6D). Furthermore, high-glucose promoted expressions of pyroptosis associated proteins (Caspase-1, Gasdermin D, NLRP3, IL-1β and IL-18) in RPE cells, which were reversed by overexpressing METTL3 (Figure 6E, 6F). Of note, the alleviating effects of overexpressed METTL3 on high-glucose induced RPE cell pyroptosis were also abrogated by upregulating PTEN (Figure 6E, 6F).

## DISCUSSION

Diabetic retinopathy (DR) is a common microvascular complication of diabetes [33], considered as the main cause of diabetes related blindness worldwide, and seriously endangering to human health [34]. However, due to its complicated pathogenesis and unknown mechanisms, there are still no effective therapies for DR treatment in clinic [35]. Recent studies found that methyltransferase-like 3 (METTL3) mediated m^6^A modifications were closely related with the development of type 2 diabetes mellitus (T2DM) [6], but it was still unclear whether METTL3 regulated DR progression. The high-glucose treated retinal pigment epithelium (RPE) cells were used in this study as the *in vitro* models for DR research according to the previous studies [36–38]. The results showed that METTL3 was low expressed in the peripheral venous blood samples collected from T2DM patients compared to the normal volunteers. In addition, high-glucose inhibited METTL3 expressions in RPE cells, indicating that METTL3 might participate in the regulation of DR progression. Further results validated that the promoting effects of high-glucose on RPE cell apoptosis and pyroptosis were reversed by overexpressing METTL3 and aggravated by knocking down METTL3. The above results suggested that high-glucose inhibited RPE cell viability by downregulating METTL3.

MicroRNAs (miRNAs) involved in the regulation of DR progression and RPE cell functions [39, 40], and miRNAs could be regulated by METTL3 in a m^6^A modifications dependent manner [17]. By screening the potential downstream miRNAs (let-7e, miR-221/222, miR-4485, miR-25-3p, miR-93, miR-126 and miR-335) of METTL3 [4, 18], we found that high-glucose specifically decreased the levels of miR-25-3p, instead of other miRNAs, in RPE cells. Further experiments verified that METTL3 overexpression increased miR-25-3p levels in RPE cells, which were abrogated by knocking down the microprocessor protein DGCR8 and indicated that METTL3 regulated miR-25-3p in RPE cells through DGCR8. Besides, miR-25-3p was low-expressed in the clinical samples of T2DM patients compared to their normal counterparts. In addition, previous publication reported that overexpression of miR-25-3p inhibited high-glucose induced apoptosis in renal tubular epithelial cells [31], which were also validated in RPE cells in this study. Specifically, the effects of high-glucose on RPE cell proliferation, apoptosis and pyroptosis were reversed by overexpressing miR-25-3p and enhanced by knocking down miR-25-3p. The above results indicated that high-glucose inhibited RPE cell viability by downregulating miR-25-3p and in line with the previous study [31]. Interestingly, this study found that the protective effects of METTL3 overexpression on high-glucose induced RPE cell death were abrogated by knocking down miR-25-3p, implying that high-glucose inhibited RPE cell viability by regulating METTL3/miR-25-3p signaling cascade.

Previous studies reported that PTEN/Akt signal pathway participated in the regulation of diabetes mellitus [25, 26] and DR progression [27], and overexpressed miR-25-3p alleviated high-glucose induced renal tubular epithelial cell death by regulating PTEN/Akt signal pathway [31], which were also verified in this study in RPE cells. Mechanistically, high-glucose promoted PTEN, while inhibited phosphorylated Akt expressions in RPE cells, which were all reversed by overexpressing miR-25-3p. In addition, we found that overexpressed METTL3 decreased the expression levels of PTEN, and promoted phosphorylated Akt expressions in RPE cells, which were all reversed by knocking down miR-25-3p. The above results indicated that high-glucose regulated PTEN/Akt signal pathway by downregulating METTL3 and miR-25-3p in RPE cells. Furthermore, the protective effects of overexpressed METTL3 on high-glucose induced RPE cell death were abrogated by overexpressing PTEN, which indicated that overexpressed METTL3 alleviated the cytotoxic effects of high-glucose on RPE cells by downregulating PTEN.

Taken together, this study found that overexpression of METTL3 alleviated high-glucose induced RPE cell apoptosis and pyroptosis, and promoted cell proliferation by regulating miR-25-3p/PTEN/Akt signaling cascade in a DGCR8-dependent manner. This study uncovered the underlying mechanisms of DR pathogenesis, and will shed light on the discovery of potential therapeutic agents for DR treatment in clinic.

## MATERIALS AND METHODS

### Clinical specimens

The patients (N=30) diagnosed with type II diabetes mellitus (T2DM) and healthy volunteers (N=30) were recruited in the 2^nd^ Affiliated Hospital of Kunming Medical University from 2014 to 2016. The peripheral venous blood samples were collected from the above participants and immediately frozen in the refrigerator with 4 °C for further experiments. The T2DM patients were chosen in this study according to the criteria of the American Diabetes Association [41]. All the participants had signed the informed consent form. Besides, all the clinical experiments in this study were conducted in accordance with the Declaration of Helsinki, and approved by the Ethics Committee of 2^nd^ Affiliated Hospital of Kunming Medical University.

### Cell culture and vectors transfection

The human retinal pigment epithelium (RPE) cell line ARPE-19 was obtained from the American Type Culture Collection (ATCC, USA). The ARPE-19 cells were cultured in the Dulbecco’s modified Eagle’s medium (DMEM, Gibico, USA) containing 10% fetal bovine serum. The cells were then put into the incubator with humidified atmosphere containing 5% CO_2_ at 37°C. The miR-25-3p mimic and inhibitor were designed and synthesized by Sangon Biotech (Shanghai, China). The small interfering RNA for METTL3 was obtained from RiboBio (Guangzhou, China). The cDNA fragments for METTL3 and PTEN were amplified and cloned into pcDNA3.1 vectors to obtain overexpressed vectors for METTL3 (OE-METTL3) and PTEN (OE-PTEN), respectively, which were constructed by Sangon Biotech (Shanghai, China). Finally, the Lipofectamine 2000 transfection kit (Invitrogen, USA) was employed to deliver all the above vectors into ARPE-19 cells according to the manufacturer’s instruction.

### Real-Time qPCR

The TRIzol kit (Invitrogen, USA) was employed to extract the total RNA from ARPE-19 cells according to its protocol. Besides, the total RNA from the clinical samples were prepared according to the previous study [39]. The iScript cDNA Synthesis Kit (Bio-rad, USA) was used to reversely transcribed the RNA into cDNA, and HiScript II Q Select RT SuperMix (Vazyme, China) was employed to quantify the expression status of the target genes. The primer sequences for Real-Time qPCR were listed in Table 1.

**Table 1:** The primer sequences for Real-Time qPCR.

**Gene**	**Primer sequences (strand)**
β-actin	Forward: 5’-CTCCATCCTGGCCTCGCTGT-3’ Reverse: 5’-GCTGCTACCTTCACCGTTCC-3’
U6	Forward: 5’-GACTATCATATGCTTACCGT-3’ Reverse: 5’-GGGCAGGAAGAGGGCCTAT-3’
miR-25-3p	Forward: 5’-CTCCCTCACAGGACAGCTGAACAC-3’ Reverse: 5’-CTGCCCCCCCACATCTGCAGT-3’
METTL3	Forward: 5’-TTGTCTCCAACCTTCCGTAGT-3’ Reverse: 5’-CCAGATCAGAGAGGTGGTGTAG-3’

### Western blot

The RIPA lysis buffer (Beyotime, China) was used to extract the total proteins from the ARPE-19 cells according to the manufacturer’s protocol. The protein concentrations were determined by using the BCA protein assay kit (Beyotime, China). After that, the proteins were separated by 10% SDS-PAGE and transferred onto PVDF membranes (Millipore, USA). The PVDF membranes were then blocked by 5% skim milk for 60 min at room temperature and probed with the primary antibodies against β-actin (Abcam, UK), METTL3 (Abcam, UK), Caspase-1 (Abcam, UK), Gasdermin D (Abcam, UK), NLRP3 (Abcam, UK), IL-1β(Abcam, UK), IL-18 (Abcam, UK), Cyclin D1 (Abcam, UK), CDK2 (Abcam, UK), Cyclin E2 (Abcam, UK), p27 (Abcam, UK), Bax (Abcam, UK), cleaved Caspase-3 (Abcam, UK), Bcl-2 (Abcam, UK), PTEN (Abcam, UK), p-Akt (Abcam, UK) and Akt (Abcam, UK) overnight at 4°C. The secondary antibody (Abcam, UK) was then incubated with the membranes for 2h at room temperature. Finally, the protein bands were visualized by ECL Western Blot detection kit (GE Healthcare Bio-science, USA) and quantified by Image J software.

### Cell counting kit-8 (CCK-8) assay

The ARPE-19 cells were harvested and seeded into the 96-well plates at the density of 2 × 10^3^ per well. The high-glucose (50mM) were then incubated with the cells for 0h, 12h, 24h and 36h, respectively. The commercial CCK-8 kit (AbMole, USA) was employed to measure cell proliferation according to the manufacturer’s protocol. Briefly, 10 μl of CCK-8 solution was added into each well for 4 h. After that, the plates were gently mixed and the Gemini EM microplate reader (Molecular Devices, USA) was used to measure the optical density (OD) values at the absorbance of 450 nm. The OD values were used to reflect the proliferation abilities of ARPE-19 cells.

### Enzyme-linked immunosorbent assay (ELISA)

The supernatants for ARPE-19 cells were collected and the commercial ELISA kit (Peprotech, USA) was used to measure the expression levels of IL-1β and IL-18 according to the manufacturer’s instruction. The HRP-labeled goat anti-rabbit IgG antibodies were used as secondary antibodies in this study. The microplate reader (Molecular Devices, USA) was used to detect the absorbance values at the wavelength of 450 nm.

### Flow cytometry (FCM)

The ARPE-19 cells were transfected with the above vectors and treated with high-glucose (50 mM) for 36h, the cell apoptosis ratio was determined by using the Annexin V-FITC/Propidium Iodide (PI) Apoptosis Detection Kit (BD Biosciences, USA) according to the manufacturer’s instruction. In brief, the staining solutions for Annexin V and PI were incubated with the cells for 30 min at darkness. The Flow cytometry (FCM) produced by ThermoFisher Scientific (USA) was used to measure cell apoptosis ratio.

### Colony formation assay

The ARPE-19 cells were transfected with the above vectors and treated with high-glucose (50mM) for 36 h. The colony formation assay was conducted to evaluate cell proliferation ability. The ARPE-19 cells were harvested and cultured in the 6-well plates at the density of 500 cells per well for 14 days and stained with crystal violet (Beyotime, China). The cell colonies containing at least 10 cells were counted by using an inverted microscope (ThermoFisher Scientific, USA).

### Dual-luciferase reporter gene system

The wild-type (Wt) and mutant-type (Mut) 3’ UTR regions of PTEN mRNA were cloned into the luciferase expressing pMIR-REPORT vector (ThermoFisher). The above vectors were co-transfected with mimic and inhibitor for miR-25-3p, and miR-NC into HEK-293T cells by using the Lipofectamine 2000 transfection kit (Invitrogen, USA). The commercial dual-luciferase reporter assay kit (Promega, USA) was employed to measure the relative luciferase activity, which were quantified by the luminescence plate reader (Molecular Devices Inc., USA).

### Statistical analysis

All the data in our study were collected and represented as Means ± Standard Deviation (SD). The SPSS 13.0 software was used to analyze the data. The comparisons between two groups were conducted by using the Student’s t-test. The comparisons among multiple groups were conducted by using the one-way analysis of variance (ANOVA). The correlation between miR-25-3p and METTL3 mRNA in clinical serum samples were analyzed by employing the Pearson Correlation Analysis. All the experiments in this study were repeated at least 3 times. “*p* < 0.05” means statistical significance.
